# Specialist oncological surgery for removal of the ovaries and fallopian tubes in *BRCA1* and *BRCA2* pathogenic variant carriers may reduce primary peritoneal cancer risk to very low levels

**DOI:** 10.1002/ijc.33378

**Published:** 2020-11-11

**Authors:** Emma J. Crosbie, Nicola Flaum, Elaine F. Harkness, Richard D. Clayton, Cathrine Holland, Pierre Martin‐Hirsch, Nick Wood, Patrick Keating, Emma R. Woodward, Fiona Lalloo, Paul Donnai, Richard J. Edmondson, D. Gareth Evans

**Affiliations:** ^1^ Division of Cancer Sciences, School of Medical Sciences, Faculty of Biology, Medicine and Health University of Manchester, Manchester Academic Health Science Centre Manchester UK; ^2^ Department of Obstetrics and Gynaecology St Mary's Hospital, Manchester University NHS Foundation Trust Manchester UK; ^3^ Division of Evolution and Genomic Sciences School of Biological Sciences, Faculty of Biology, Medicine and Health, University of Manchester, Manchester Academic Health Science Centre Manchester UK; ^4^ Genetic Medicine, Manchester Centre for Genomic Medicine and NW Laboratory Genetics Hub Manchester University Hospitals NHS Foundation Trust Manchester UK; ^5^ Division of Informatics, Imaging and Data Sciences, School of Health Sciences, Faculty of Biology Medicine and Health, University of Manchester Manchester UK; ^6^ Prevention Breast Cancer Centre and Nightingale Breast Screening Centre University Hospital of South Manchester Manchester UK; ^7^ Department of Obstetrics and Gynaecology Royal Preston Hospital, Lancashire Teaching Hospitals NHS Trust Preston UK; ^8^ The Christie NHS Foundation Trust Manchester UK; ^9^ Manchester Breast Centre, Manchester Cancer Research Centre University of Manchester Manchester UK

## Abstract

Risk‐reducing bilateral salpingo‐oophorectomy (RRBSO) is highly effective for the prevention of high‐grade serous ovarian cancer (HGSOC) in *BRCA1/2* pathogenic variant carriers (PVCs), but does not completely eliminate future risk of primary peritoneal cancer (PPC). The requirement to completely remove fallopian tubes at RRBSO and carefully exclude occult cancer/serous tubal intraepithelial carcinoma (STIC) lesions may not have been appreciated historically. We calculated rates of HGSOC and PPC in confirmed *BRCA1/2* PVCs registered on the regional database in those who did (cases) and did not (controls) undergo RRBSO after genetic testing. Expected annual rates of ovarian/peritoneal cancer were 1% for *BRCA1* ≥ 35 years and 0.5% for *BRCA2* ≥ 45 years. Follow‐up before 35/45 years was “risk free” and lead time excluded RRBSO <35 years and <45 years for *BRCA1* and *BRCA2*, respectively. Women were followed from personal mutation report (controls) or RRBSO (cases) to death, ovarian/peritoneal cancer or last follow‐up, whichever was sooner. In total, 891 cases (*BRCA1* = 468, *BRCA2* = 423) and 1302 controls had follow‐up ≥35 years (*BRCA1* = 736) and ≥45 years (*BRCA2* = 566), respectively, over a total of 7261.1 risk eligible years (mean = 8.15 years). Twenty‐one occult ovarian cancers were found at RRBSO (2.4%), 16 at stage 1. Post RRBSO, 56.97 ovarian/peritoneal cancers were expected but only 3 were observed (HR = 0.053; 95% CI = 0.013‐0.14), with combined Kaplan‐Meier analysis HR = 0.029 (95% CI = 0.009‐0.100, *P* < .001). Risk reduction was greater in specialist (HR = 0.03; 95% CI = 0.001‐0.13) compared to non‐specialist centres (HR = 0.11; 95% CI = 0.02‐0.37) (*P* = .07). In controls, 23.35 ovarian/peritoneal cancers were expected with 32 observed (HR = 1.37; 95% CI = 0.95‐1.91). RRBSO <35/<45 years reduces the risk of ovarian/peritoneal cancer by 95% in *BRCA1/2* PVCs and may be greater in specialist centres.

## Abstract 2

**What's new?**

Women who carry genetic variants in *BRCA1/2* that are linked to high‐grade serous ovarian cancer are advised to undergo risk‐reducing prophylactic bilateral salpingo‐oophorectomy (RRBSO). RRBSO, however, is associated with a significant residual risk of primary peritoneal cancer (PPC). Here, the rate of PPC following prophylactic surgery for high‐grade serous ovarian cancer was investigated among 2,193 *BRCA1/2* mutation carriers who had undergone RRBSO. Estimates indicate that prophylactic surgery reduced the overall risk of PPC by 95 percent, a far greater reduction than previously described. Residual risk was lowest when RRBSO was carried out at specialist centers compared to non‐specialist centers.

**Abbreviations**
HGSOC: high‐grade serous ovarian cancer
HR: hazard ratio
O:E: observed to expected
PARP: poly‐ADP ribose polymerase
PPC: primary peritoneal cancer
PVC: pathogenic variant carrier
RRBSO: risk‐reducing bilateral salpingo‐oophorectomy
SEE‐FIM: sectioning and extensively examining the fimbriated end
STIC: serous tubal in‐situ carcinoma

## 1 INTRODUCTION

High‐grade serous ovarian cancer (HGSOC) is a strongly heritable cancer, with a 3‐fold increase in risk of developing the disease in women with first‐degree relatives with ovarian cancer.^1^ Carriers of germline pathogenic variants (PVs) in *BRCA1* or *BRCA2* have a high lifetime risk of ovarian cancer, in particular high‐grade serous pathology.^2^ Women with PVs in *BRCA1* or *BRCA2* have a cumulative lifetime risk of ovarian cancer of 44% to 61% and 17% to 24%, respectively.^3^, ^4^, ^5^ As early detection of ovarian cancer using serum CA125 and transvaginal ultrasound scans has not been effective at reducing sufficient cancers to early stage (1‐2),^6^, ^7^ and cancers detected on such screening are still associated with high mortality,^8^ carriers of PVs in these genes are strongly advised to undergo risk‐reducing bilateral salpingo‐oophorectomy (RRBSO).^9^ This surgery is usually encouraged at or just before the main ovarian cancer risk period starts at 35 to 40 years for *BRCA1* and 40 to 45 years for *BRCA2*.^10^ Female carriers of PVs in *BRCA1/2* undertaking RRBSO have increased life expectancy mainly due to reduction in ovarian cancer risk, although there may be some reduction in breast cancer risk and reduced mortality from previous breast cancer.^11^, ^12^, ^13^

Women undergoing RRBSO are warned of a residual risk of primary peritoneal cancer (PPC), which was first described in 1982.^14^ A meta‐analysis of studies assessing this risk suggested only a 79% reduction in risk of an “ovarian” type cancer after RRBSO in *BRCA1/2* PV carriers^15^ (hazard ratio = 0.21; 95% CI = 0.12‐0.39). Subsequent review suggested that this residual risk may be mitigated by earlier surgery and was predominantly seen in *BRCA1* PV carriers.^16^ The origin of PPC has been hypothesised to be due to one of three sources. Firstly, “ovarian rest cells” displaced during the embryological journey in the abdominal cavity may be a primary origin. Secondly, cells from the fimbria/ovaries could be displaced during adulthood into the peritoneum or thirdly through dissemination at the time of RRBSO. The latter two possibilities have become more prominent since the description of serous tubal in‐situ carcinoma (STIC) lesions in the fimbrial end of the fallopian tube as the probable precursor lesion for most high‐grade serous ovarian/tubal cancers.^17^ Indeed, the presence of STIC lesions was predictive of future PPC in one single institution study with 2 of 7 (28.6%) developing subsequent PPC compared to only 1 of 287 (0.3%) without STIC.^18^ The theory regarding potential prevention of ovarian/tubal cancers by removal of the fallopian tubes has resulted in a number of pilot studies to assess the potential benefits of early tubal surgery and delayed oophorectomy to mitigate the effects of early surgical menopause on subsequent health and quality of life.^19^, ^20^

We have previously published an early series of RRBSO where we noted that there were no cases of PPC following 300 surgeries that included 160 *BRCA1/2* PV carriers.^21^ The recent development of a second PPC in a *BRCA2* PV carrier operated on outside a gynaecological oncology centre prompted us to revisit the PPC risk in 891 *BRCA1/2* PV carriers who have tested positive in our region and have undergone RRBSO.

## 2 PATIENTS AND METHODS

Female carriers of PVs in *BRCA1* or *BRCA2* were identified from our regional register covering a population of 5 million in Northwest England as previously described.^10^, ^11^ A total of 3653 female PV carriers were identified from 1753 families. Women were eligible if they had undergone RRBSO without any evidence on CA125 and ultrasound of the prior presence of ovarian cancer. The controls were women who had not undergone RRBSO including any time post genetic testing before RRBSO (to avoid bias of not including this follow‐up). Cases were followed from date of RRBSO to date of death, PPC or date of last follow‐up, whichever was earlier. Controls were followed from date of personal mutation report to date of death, ovarian/peritoneal cancer or date of last follow‐up, whichever was earlier. Cases were censored at date of surgery if ovarian cancer was identified as an occult lesion. As we were not aware of any PPC cases postsurgery in any of our *BRCA1/2* families and all RRBSO were recorded in the specialty centres for high‐risk women since 1980,^21^, ^22^ we included follow‐up from surgery in those identified as PV carriers after RRBSO.

### 2.1 Ovarian/tubal/peritoneal primary risk

We chose a conservative estimate of ovarian cancer risk based on the recent prospective series showing a 44% and 17% risk for *BRCA1* and *BRCA2*, respectively, to age 80 years.^4^ This is lower than our previous in‐house estimates.^10^, ^21^ Risk for *BRCA1* was considered at 1% annual risk from age 35 years (45% risk to age 80 years) and 0.5% risk for *BRCA2* from age 45 years (17.5% risk to age 80 years). Follow‐up before 35 years was considered to be “risk free” and calculation of “lead time” excluded RRBSO before the risk period for both genes and <45 years for *BRCA2*. We also performed an analysis using 5‐year cumulative risks from a typical pedigree in BOADICEA v.3 (https://pluto.srl.cam.ac.uk/cgi-bin/bd3/v3/bd.cgi), but this substantially underestimated risk in our control population *BRCA1* (expected 14.48; observed 22 O:E = 1.49; *P* = .09) *BRCA2* (expected 4.47; observed 10 O:E = 2.24; *P* = .033). In fact 5‐year risks for ovarian cancer did not change with different family histories (supplementary Table 1). The model particularly underestimated risk in *BRCA1* >50 years (expected 6.98; observed 15 O:E = 2.15; *P* = .011) and in *BRCA2* > 60 years (expected 0.89; observed 6 O:E = 6.74; *P* < .001). We therefore chose to continue with our life table approach.

### 2.2 Types of surgery and centre designation

Operations undertaken at two designated gynaecological cancer centres at St Mary's Hospital Manchester and Royal Preston Hospital were considered specialty surgeries. RRBSO carried out in other units in the region was considered nonspecialty. From 1980 to 2008, the predominant RRBSO procedure was a total abdominal hysterectomy and RRBSO.^21^, ^22^ This involved “bagging” of the ovaries and fallopian tubes at surgery with peritoneal lavage after surgical removal.^21^ Since 2009, the predominant procedure has been laparoscopic RRBSO without hysterectomy. This involves complete removal of all ovarian tissue and the full length of both fallopian tubes using specimen retrieval bags. Since 2011, careful pathological examination of the fallopian tubes has included an assessment for STIC lesions by specialist gynaecological pathologists in specialty centres using the SEE‐FIM protocol. The surgical procedures in nonspecialty hospitals did not follow standardised protocols (eg, “bagging” of tubes and ovaries) or submit surgical specimens for specialist gynaecological pathology review.

### 2.3 Statistics

Number and percent are reported for categorical variables with differences assessed by the Chi square or Fisher exact test where appropriate. For continuous variables, such as age at surgery, we report the range, mean and median. Lead time was estimated as the proportion of occult cancers of those censored at RRBSO multiplied by the estimated annual risk for ovarian cancer (1% in *BRCA1*, 0.5% in *BRCA2*). Expected numbers were obtained by multiplying follow‐up times by the estimated annual risks for each PV carrier and observed to expected ratios with 95% confidence intervals were calculated. Cumulative incidence curves were obtained using Kaplan‐Meier curves from date of individual genetic test to date of RRBSO or censor (ovarian cancer, death, or last follow‐up) and from date of RRBSO to PPC or censor (death or last follow‐up). Follow‐up of less than 6 months from RRBSO was set at zero. All *P* values were based on two‐sided tests and were considered statistically significant if <0.05. Analyses were performed in Stata version 14 (StataCorp. 2015. Stata Statistical Software: Release 14. College Station, TX: StataCorp LP).

## 3 RESULTS

**FIGURE 1:**
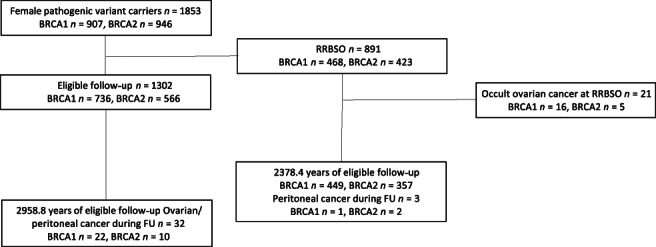
Study flowchart. RRBSO, risk reducing bilateral salpingo‐oophorectomy; FU, follow‐up

A total of 891 proven *BRCA1/2* PV carriers (*BRCA1* = 468, *BRCA2* = 423) who had undergone RRBSO were identified with a median age of 45.1 at RRBSO (Figure 1, Table 1). Of the *BRCA1* carriers, 236 (50.4%) had prior or prospective breast cancer with 201 (43%) having chemotherapy. For *BRCA2* carriers, 230 (54.4%) had prior or prospective breast cancer with 179 (42.3%) having chemotherapy. Overall, 1853 controls were identified with a mean age of 44.8 at mutation report (median = 43.45). Of those never having undergone RRBSO, a slightly higher proportion of controls had breast cancer (*BRCA1* 60.1%, *BRCA2* 60.4%) and chemotherapy (51.4%/45.4%) (Table 2). There were only 1302 who had follow‐up that was eligible after age 35 years for *BRCA1* (n = 736) and 45 years for *BRCA2* (n = 566) (Table 3), this included 539 women (mean age at mutation 46.9; median 46.3) who later had RRBSO. Cases underwent surgery aged 24.9 years to 79.3 years (median = 45.1 years) and were 27.9 years to 88.1 years at censoring (median = 53.8). Four hundred of 891 (44.9%) had RRBSO after a breast cancer diagnosis (range 0.4‐38.6 years post diagnosis; mean 6.8 years; median 4.6 years; 114 within 2 years of breast cancer). Most women (689/891‐77%) had RRBSO after their genetic test result (range = 0.016‐19.8 years; mean = 2.3 years; median = 0.83 years). Of the 104 (15%) delaying RRBSO more than 5 years post report (*BRCA1* = 57), the mean age at testing was 36.7 years (median = 34.99; range 22.3‐59.4 years), with 27 aged <31 years. The proportion without children in each age range at RRBSO is shown in Table 4. There was no strong evidence for a higher proportion of nulliparous women undergoing RRBSO >40 years of age with 12.2% <40 vs 14% aged ≥40 (*P* = .57).

**TABLE 1:** Follow‐up and expected cancers in 891 *BRCA1/2* PV carriers who had undergone RRBSO

				Total RRBSO				TAH BSO				BSO			
Centre/ unit	Gene	Number	Median age at RRBSO (IQR)	Total follow‐up from RRBSO (median/mean)	Eligible follow‐up	Expected OC	PPC	Number with TAH BSO	Eligible follow‐up TAH BSO	Expected OC	PPC	Number with BSO only	Eligible follow‐up BSO only	Expected OC	PPC
St Mary's	*BRCA1*	263	42.6 (38.6‐48.1)	2551.27 (8.5/9.7)	2492.90	24.93	0	117	1621.83	16.22	0	146	871.07	8.71	0
	*BRCA2*	226	46.3 (40.5‐53.5)	2011.38 (7.6/8.9)	1737.02	8.69	1	88	969.29	4.85	0	138	767.73	3.84	1
Preston	*BRCA1*	46	43.5 (37.8‐53.8)	398.86 (8.1/8.7)	383.03	3.83	0	18	199.53	2.00	0	28	183.51	1.84	0
	*BRCA2*	38	48.8 (44.4‐53.7)	290.15 (7.9/7.6)	269.75	1.35	0	6	75.75	0.38	0	32	193.99	0.97	0
Total specialty		573		5251.66	4882.70	38.79	1	229	2886.40	23.44	0	344	2016,31	15.35	1
Other	*BRCA1*	159	45.0 (39.9‐51.6)	1268.57 (6.4/8.0)	1235.11	12.35	1	128	1083.02	10.83	1	31	152.09	1.52	0
	*BRCA2*	159	48.1 (43.4‐52.8)	1294.83 (6.5/8.1)	1143.29	5.72	1	119	955.02	4.78	1	40	188.27	0.94	0
Total other		318		2563.40	2378.40	18.07	2	247	2038.04	15.61	2	71	340.37	2.46	0
Overall total		891	45.1 (40.0‐52.3)	7815.06 (7.1/8.7)	7261.10	56.86	3	476	4904.43	39.04	2	415	2356.67	17.82	1

Abbreviations: IQR, interquartile range; OC, ovarian cancer; PPC, primary peritoneal cancer; RRBSO, risk‐reducing bilateral salpingo‐oophorectomy; TAH BSO, total abdominal hysterectomy and bilateral salpingo‐oophorectomy.

**TABLE 2:** Demographics and potential risk factors in women undergoing or not undergoing RRBSO

Ovarian cancer or PPC	Number	Mean age at RRBSO or mutation report if no RRBSO	Range	Age at ovarian cancer	Range	Nulliparous	%	Breast cancer	%	Chemotherapy for prior breast cancer	% with ovarian cancer	Ovarian family history	
*BRCA1* RRBSO	17	51.7	38.7‐73.3	52.0	38.7‐73.3	1	6%	10	58.82%	7	41.18%	8	47.06%
*BRCA2* RRBSO	7	57.0	45.4‐62.6	58.9	45.4‐72.1	0	0%	6	85.71%	3	42.86%	2	28.57%
*BRCA1* no RRBSO	22	52.7	30.7‐66.3	55.6	37.7‐68.0	3	14%	16	72.73%	12	54.55%	10	45.45%
*BRCA2* no RRBSO	10	59.8	43.4‐68.5	61.6	49.7‐70.6	0	0%	7	70.00%	4	40.00%	4	40.00%
No ovarian cancer													
*BRCA1* RRBSO	451	44.9	24.8‐76.3	N/a	N/a	67	15%	226	50.11%	194	43.02%	242	53.66%
*BRCA2* RRBSO	416	48.1	28.4‐79.3	N/a	N/a	54	13%	223	53.61%	176	42.31%	124	29.81%
*BRCA1* no RRBSO	544	42.8	18.1‐89.5	N/a	N/a	134	25%	324	59.56%	279	51.29%	201	36.95%
*BRCA2* no RRBSO	624	46.6	19.1‐89.5	N/a	N/a	122	20%	376	60.26%	284	45.51%	114	18.27%

Abbreviation: RRBSO, risk‐reducing bilateral salpingo‐oophorectomy.

**TABLE 3:** Follow‐up and observed and expected cancers in controls postgenetic testing

	Number	Number with eligible follow‐up	Eligible follow‐up (mean)	Expected OC	OC no RRBSO	OC @ RRBSO	Stage 1	PPC post‐RRSBSO	Censored at RRBSO	RRBSO before eligible age	Estimated lead time from OC at RRBSO
*BRCA1*	907	736	1709.66 (2.31)	17.10	22	16	12	1	346	18	2.89
*BRCA2*	946	566	1249.18 (2.21)	6.25	10	5	4	2	193	129	2.07
Total	1853	1302	2958.84	23.35	32	21	16	3	539	147	

Abbreviations: RRBSO, risk‐reducing bilateral salpingo‐oophorectomy; OC, ovarian cancer.

**TABLE 4:** Proportion nulliparous at each age range at RRBSO

Gene	*BRCA1*	*BRCA2*
RRBSO <35 years	33	13
Number nulliparous	6	3
%	18.18%	23.08%
RRBSO 35 to 39 years	114	61
Number nulliparous	17	1
%	14.91%	1.64%
RRBSO 40 to 44 years	123	98
Number nulliparous	20	21
%	16.26%	21.43%
RRBSO 45 to 49 years	77	92
Number nulliparous	9	12
%	11.69%	13.04%
RRBSO ≥50 years	122	160
Number nulliparous	15	17
%	12.30%	10.63%

Abbreviation: RRBSO, risk‐reducing bilateral salpingo‐oophorectomy.

### 3.1 Ovarian cancers and PPC in follow‐up and at surgery

There were 7815.1 women‐years (mean = 8.7; median = 7.1) of follow‐up to censoring from RRBSO date but only 7261.1 risk eligible years (mean = 8.15 years) (Table 1). RRBSOs occurred from 1980 to 2019 (median 2010) with 0.1 to 40 years eligible follow‐up. Only three PPCs occurred, two in women undergoing RRBSO in nonspecialty units. Of 105 RRBSO since 2011, only two STIC lesions have been identified, and a further 4 with benign ovarian cystadenoma. Twenty‐one occult ovarian cancers were found at RRBSO (2.4%) aged 38.7 to 73.3 years (median = 51.9 years). Sixteen were diagnosed at Stage 1 (76%) including one STIC lesion with microinvasion, one at Stage 2 and two each at Stages 3 and 4 (despite normal ovarian screening). One was a clear cell carcinoma, one a granulosa cell tumour and the remainder were HGSOC. Sixteen of the occult tumours occurred in *BRCA1* PV carriers and five in *BRCA2* carriers. Four hundred thirty‐five RRBSO were carried out in the risk period in *BRCA1* PV carriers and 252 in *BRCA2* PV carriers. Using an annual incidence rate of 1% for *BRCA1* and 0.5% for *BRCA2*, this would suggest lead times of 3.7 years for *BRCA1* and 3.9 years for *BRCA2*. However, confining analysis to only those who were known carriers at RRBSO, this drops to 2.89 and 2.07 years, respectively. There were 63 breast cancers post RRBSO (range 0.1‐38.8 years post RRBSO; mean‐7.4 years; median 6.26 years).

### 3.2 Expected ovarian cancers

A total of 56.97 ovarian cancers were expected using life tables (Table 1) with only three PPCs occurring (HR = 0.053 95% CI = 0.013‐0.14), with a rate of 0.4 per 1000 women‐years. Within the designated cancer centres, 38.79 were expected with one cancer observed (HR = 0.03 95% CI = 0.001‐0.13) compared to 18.07 expected and 2 observed in the nonspecialty units (HR = 0.11 95% CI = 0.02‐0.37) (*P* = .07). In the control group, there were 2958.8 years of eligible follow‐up in 1302 women (mean = 2.3; median = 0.92; range 0.01‐43.2). Controls eligible for follow‐up had a mean age of 50.3 (median 48.4) with those undergoing RRBSO, a mean age of 47.5 (median 46.3). The annual incidence of ovarian cancer was 10.8 per 1000 (odds ratio for RRBSO = 0.037, 95% CI 0.002‐0.342). There were 17.1 ovarian cancers expected with 22 observed in *BRCA1* PV carriers, and 6.25 expected and 10 observed in *BRCA2* PV carriers. The overall difference among controls was not significant (HR = 1.37; 95% CI = 0.95‐1.91). This provided an observed:expected ratio of 1.28 and 1.6, respectively, for *BRCA1* and *BRCA2* genes. If these were extrapolated to the post RRBSO follow‐up, the expected cancers would rise to 52.8 for the specialty centres.

Two PPCs occurred after hysterectomy and RRBSO procedures, one aged 56.0 years (4.2 years post‐surgery) following a vaginal hysterectomy with abdominal‐assisted RRBSO in a *BRCA1* carrier and one aged 72.1 years (9.5 years postsurgery) in a *BRCA2* carrier. The third was in a laparoscopic RRBSO at St Mary's 3.9 years postsurgery in a *BRCA2* carrier. There were therefore two PPCs in hysterectomy cases with 38.9 expected (HR = 0.051, 95% CI = 0.01‐0.17) with one in RRBSO only surgery with 17.65 expected (HR = 0.06; 95% CI = 0.003‐0.28).

**FIGURE 2:**
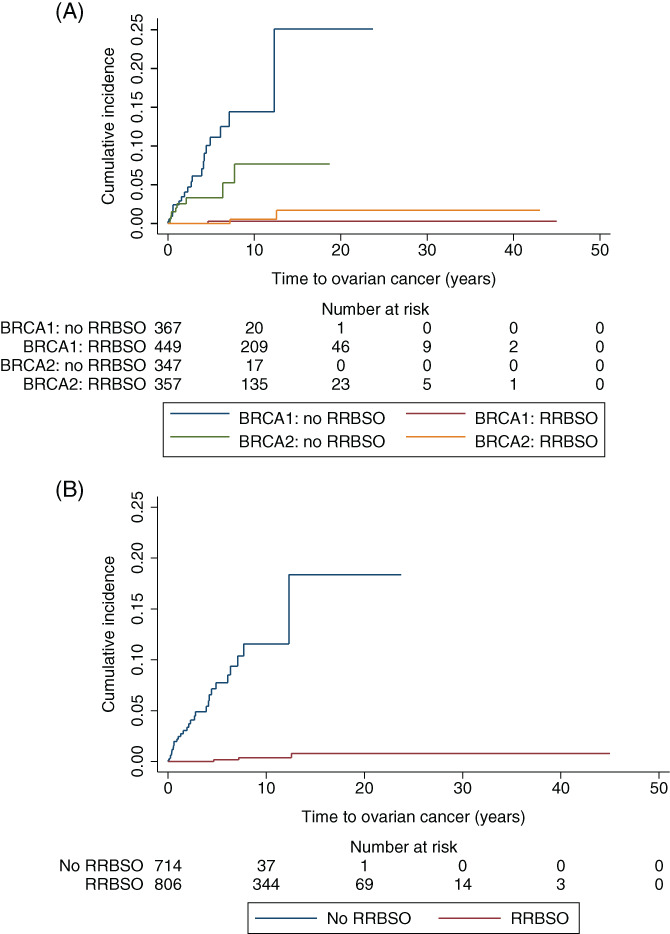
Kaplan–Meier analysis comparing ovarian/peritoneal cancer incidence in women undergoing RRBSO vs no RRBSO. A, RRBSO was associated with significant risk reduction for ovarian/peritoneal cancers for both *BRCA1* (HR 0.014, 95% CI = 0.002‐0.106, *P* < .001) and *BRCA2* pathogenic variant carriers (HR 0.072, 95% CI = 0.014‐0.366, *P* = .002). B, RRBSO was associated with reduced risk of ovarian/peritoneal cancers when considering *BRCA1* and *BRCA2* pathogenic variant carriers combined (HR 0.029, 95% CI = 0.009‐0.100, *P* < .001)

We next performed Kaplan‐Meier analysis comparing RRBSO vs no RRBSO (Figure 2A,B). For *BRCA1*, RRBSO (1/449‐PPC) vs no RRBSO (21/367‐ovarian cancer), total follow‐up 5769.8 years, was associated with a HR for ovarian/peritoneal cancer of 0.014 (95% CI = 0.002‐0.106) (*P* < .001), and for *BRCA2*, RRBSO (2/357‐PPC) vs no RRBSO (9/347‐ovarian cancer), total follow‐up 4174.5 years, was associated with a HR for ovarian/peritoneal cancer of 0.072 (95% CI = 0.014‐0.366) (*P* = .002) (Figure 2A). In combined analysis of *BRCA1* and *BRCA2*, RRBSO (3/806) vs non‐RRBSO (30/714), total follow‐up 9944.3 years was associated with a HR of 0.029 (95% CI = 0.009‐0.100) (*P* < .001) (Figure 2B, Supplementary Figure 1).

Table 2 presents potential risk factors for ovarian cancer and potential motivators for RRBSO. There was no apparent effect of nulliparity on ovarian cancer risk. Women with ovarian cancer were more likely to have had a previous breast cancer (39/56‐69.6%) than women without (1149/2035‐56.5%); *P* = .055. However, chemotherapy associated with breast cancer was equally common in women with ovarian cancer (26/56‐46.4%) compared to women without (933/2035‐45.8%). Although women undergoing RRBSO were less likely to have had a previous/incident breast cancer [465/891‐52.2% vs 723/1200‐60.3% in controls (*P* = .0003)], or to have had chemotherapy [380/891‐42.6% vs 579/1200‐48.3% (*P* = .011)], women with ovarian cancer were more likely to have a family history of ovarian cancer (24/56‐42.9%) than those without (681/2035‐33.5%), but this was not significant (*P* = .15). Women who undertook RRBSO were significantly more likely (376/891‐42.2%) to have an ovarian cancer family history than those who did not (329/1200‐27.4%) (*P* < .0001).

### 3.3 Deaths in follow‐up

In the 891 RRBSO cases, there were 30 *BRCA1* carriers (6.4%) who died during follow‐up, six due to ovarian cancer, five of whom were diagnosed at RRBSO, including three Stage 1 cancers and one post RRBSO PPC. There were 14 breast cancer deaths, 2 pancreatic cancers, and 1 each of gastric and liver cancer as well as 6 noncancer deaths. Among *BRCA2* carriers, there were 34 deaths (8.0%), 1 from an occult ovarian cancer at RRBSO and 26 breast cancer deaths, 1 from melanoma, lymphoma, pancreatic, endometrial, lung and stomach cancer, plus 2 noncancer deaths. Overall, only 7 of 891 (0.78%) died from an ovarian‐related cancer and only 1 from PPC post RRBSO. Of those not having undergone RRBSO who had follow‐up in the risk period, 15 of 763 (2% age range = 39.7‐72 years; median = 60.6 years; *BRCA1* = 12) died from ovarian cancer post genetic testing. There were 108 breast cancer deaths (many had breast cancer before genetic testing) and 13 other cancer deaths likely to be unrelated to *BRCA1/2*.

### 3.4 PPC in whole series

We assessed the proportion of all ovarian cancers in the regional register who had a proven PV. There were 27 reported PPCs of 752 total ovary‐related cancers (3.6%) in *BRCA1* with age at diagnosis ranging from 39 to 81 years (median 59). This was slightly older than all ovarian cancers (range 29‐83 years, median 50.6 years). For *BRCA2*, there were 28 of 421 PPCs (6.7%—range 36‐87, median 63 years) slightly older than all *BRCA2* ovarian cancers (range 32‐89 years, median 58.0 years). These are in addition to the 3 of 56 (5.4%) presented in this report.

## 4 DISCUSSION

The current study is to our knowledge the largest single centre report on RRBSO in *BRCA1/2* PV carriers. The findings support early RRBSO ideally just before the main risk periods for ovarian cancer in *BRCA1* aged 35 years (there were two ovarian cancers aged 37.7 and 38.7 years) and *BRCA2* aged 45 years. The presence of only one PPC in our extended series of RRBSO carried out in specialty centres is encouraging because although this does not necessarily eliminate PPC risk, the remaining risk is low and almost certainly below 10% of the rate without surgery (HR = 0.03). This was supported by a combined analysis of *BRCA1* and *BRCA2* in Kaplan‐Meier analysis with RRBSO having a HR for ovarian/peritoneal cancer of 0.029 (95% CI = 0.009‐0.100, *P* < .001). The upper confidence range of a HR of 0.10 to 0.13 substantially excludes the HR of 0.21 from the meta‐analysis.^15^ Compared to a predicted risk of 8.8% to 12.6% of PPC for a 35‐year‐old *BRCA1* carrier based on only a 79% reduction in the 40% to 60% lifetime risk,^15^ the risks would be reduced to 0.4% to 7.8% using the 95% CI by reducing risks by 87% to 99%. Results from another large series of 238 *BRCA1/2* carriers found a 20‐year risk of 3.9% for *BRCA1*, which extrapolated to lifetime risk of >8%.^23^ Two of the three PPCs occurred outside the specialty centres but still represented an 89% reduction in ovarian type cancer risk in those units. Given that this study used conservative estimates of ovarian cancer incidence lower than those in our previous reports,^10^, ^21^ the findings could suggest even greater reductions in risk. However, there remains a definite risk of occult malignancy at surgery of around 3.7% in *BRCA1* and 2.4% in *BRCA2* when surgeries are undertaken during the risk period. Unfortunately while most of these cancers were detected at Stage 1, three of these cases subsequently died from their ovarian cancer. There is evidence that PARP inhibitor treatment may alter this course when used as a first‐line maintenance after primary chemotherapy.^24^ However, cancer prevention strategies through RRBSO remain the first‐line management approach, as PARP inhibitors are not curative due to the development of chemoresistance. The absence of occult lesions and postsurgical PPCs in those undergoing RRBSO before the risk period (n = 203) lends support to early RRBSO if a woman has completed her family^18^ even though this may not now reduce breast cancer risk particularly in *BRCA1* carriers.^25^, ^26^

The low level of PPC in the specialty series fits well with the concept that most PPC derives from fimbrial STIC cells that get displaced from the fimbrial ends of the fallopian tubes into the peritoneal cavity.^26^ This may even occur at the time of RRBSO if care is not taken to prevent this by careful bagging of the tubes and ovaries prior to removal from the peritoneal cavity. Furthermore, the rather poor‐risk reduction from the meta‐analysis^15^ may be due to inclusion of cases where the fallopian tubes were not removed and/or careful pathological examination of the fallopian tubes was not performed to exclude STIC lesions or occult malignancies.^27^, ^28^ Further assessment of PPC risk after RRBSO in *BRCA1/2* PV carriers where STIC lesions were identified may justify some sort of an intervention, such as staging CT scan to exclude metastatic disease or even a course of PARP inhibitor treatment if substantial rates continue to be reported.^18^ Certainly a more vigilant follow‐up may be justified. We are only aware of two STIC lesions in our series without microinvasion, and this low rate may reflect the earlier surgery particularly in more recent years with RRBSO driven by presymptomatic testing in *BRCA1/2* families.

Although the results from the meta‐analysis show only a 79% reduction in overall ovarian type cancer risk with RRBSO, some reviews have perhaps misled those counselling about risk after surgery that these are small and of the order of just 1% to 2%.^29^ The last review quoted a 1.53% incidence of PPC (28/1830); however, this represents short‐term follow‐up rather than lifetime risk.^27^ The two papers that assessed risk at 20 years came up with risks of 4.3% based on 7 PPCs post RRBSO^30^ and 3.9% in *BRCA1* carriers based on 5 PPCs.^23^ If incidence continued at the same rates, these would be equivalent to at least a risk of 8% by age 80 for a *BRCA1* carrier having surgery before 40 years of age. The present study shows convincing evidence for the first time that risks can be reduced below these levels when RRBSO is carried out in a careful, oncologically driven fashion, as the upper 95% CI excludes 8% as described earlier. Another publication that may have confused the issue was an early report on fallopian tube and peritoneal cancers that suggested risks of only 0.6% and 1.3%, respectively, based on identification of *BRCA1/2* in 5/29 (17.2%) and 9/22 (40%) of primary‐site tumours analysed.^31^ Clearly these risk estimates are incorrect for fallopian tube malignancy^27^ and also do not reflect the fact that so many *BRCA1/2* PV carriers are identified and opt for RRBSO and that PPC may be due to implantation of cells at the time of surgery.

The current study also identified a high rate of occult Stage 1 cancers at RRBSO. This suggested lead times of up to 3.9 years, although this reduced to 2 to 3 years if only those known to have a *BRCA1/2* PV at RRBSO were included. Nonetheless, this may provide hope of a reasonable sojourn time at Stage 1 to encourage more research on early detection of high‐grade serous cancers in *BRCA1/2* carriers.

There are some limitations to the present study. We do not have full details of procedures carried out beyond the two specialty centres. However, we have good long‐term follow‐up of a very large series of *BRCA1* and *BRCA2* PV carriers showing a very low rate of PPC when surgery is carried out in an oncology‐driven fashion in a specialty centre. We have not carried out a formal statistical adjustment for factors that might increase ovarian cancer risk. However, as these factors including an ovarian cancer family history and not having had chemotherapy were significantly more likely in the RRBSO group, this should mean our results are even more robust.

In conclusion, we have shown a very low rate PPC in women undergoing careful RRBSO in a specialist oncology centre. Although even longer follow‐up is required to confirm this low PPC rate especially with PPC occurring at older ages, we can already exclude the point estimate of only a 79% reduction in risk from the meta‐analysis.^15^ Therefore, the rate of PPC may be lower than previously thought if surgery is carried out early in the risk period, performed in a specialty centre and no STIC lesions identified following careful pathological assessment of entire fallopian tubes. Women undergoing such surgery should be told that this reduces their risk by 90% to 95%, and this may be more in those undergoing surgery before the risk period.

## CONFLICT OF INTEREST

RJE reports personal fees from Astra Zeneca and Arquer Diagnostics, and grants from Tesaro Inc. outside the submitted work. DGE reports personal fees from Astra Zeneca outside the submitted work. The other authors report no conflicts of interest.

## ETHICS STATEMENT

All patients provided a priori consent for their data to be used in research; data are anonymized and analysed as part of clinical audit, and so no ethical review was required.

## Supporting information


**Appendix S1.** Supporting Information.

## Data Availability

The data that support the findings of this study are available from the corresponding author upon reasonable request.
