# Supplementary material for: Specialist oncological surgery for removal of the ovaries and fallopian tubes in BRCA1 and BRCA2 pathogenic variant carriers may reduce primary peritoneal cancer risk to very low levels
Source: Int J Cancer. 2020 Nov 11;148(5):1155–63. doi: 10.1002/ijc.33378 (PMC7839461; doi:10.1002/ijc.33378)
Supplement: Supplementary file 1 — Appendix S1. Supporting Information. [file IJC-148-1155-s001.pdf]

Specialist oncological surgery for removal of the ovaries and fallopian tubes in *BRCA1* and *BRCA2* pathogenic variant carriers may reduce primary peritoneal cancer risk to very low levels.

Emma J. Crosbie, Nicola Flaum, Elaine F Harkness, Richard D. Clayton, Cathrine Holland, Pierre Martin-Hirsch, Nick Wood, Patrick Keating, Emma R. Woodward, Fiona Lalloo, Paul Donnai, Richard J. Edmondson, D. Gareth Evans

Table of contents

Supplementary Table 1……………………………………………………………………………………page 2

Supplementary Figure 1…………………………………………………………………………………page 4

Supplementary Table 1: BOADICEA 5-year ovarian cancer estimates

age	*BRCA1* (%)	*BRCA2* (%)
30-34	0.6	0.1
35-39	2	0
40-44	4.3	0.1
45-49	4.3	0.6
50-54	4.5	2.6
55-59	5.2	4.1
60-64	5.9	1.9
65-69	6.1	0.7
70-74	5.6	0.5
75-79	5.2	0.4
total	43.7	11

	BRCA1	expected BOADICEA	expected tables	Observed
30-34	351.06	0.42	N/a	
35-39	434.76	1.74	4.35	1
40-49	619.69	5.33	6.20	6
50-59	378.59	3.79	3.79	8
60-69	175.47	2.11	1.75	7
70+	101.25	1.09	1.01	0

total	2060.01	14.48	17.10	22
	BRCA2			
30-39	572.51	0.06	N/a	
40-44	363.12	0.07	N/a	
45-49	353.55	0.42	1.77	1
50-54	273.45	1.42	1.37	1
55-59	197.51	1.62	0.99	2
60-64	144.16	0.55	0.72	4
65-69	123.75	0.17	0.62	1
70+	156.79	0.16	0.78	1
total	2184.83	4.47	6.25	10

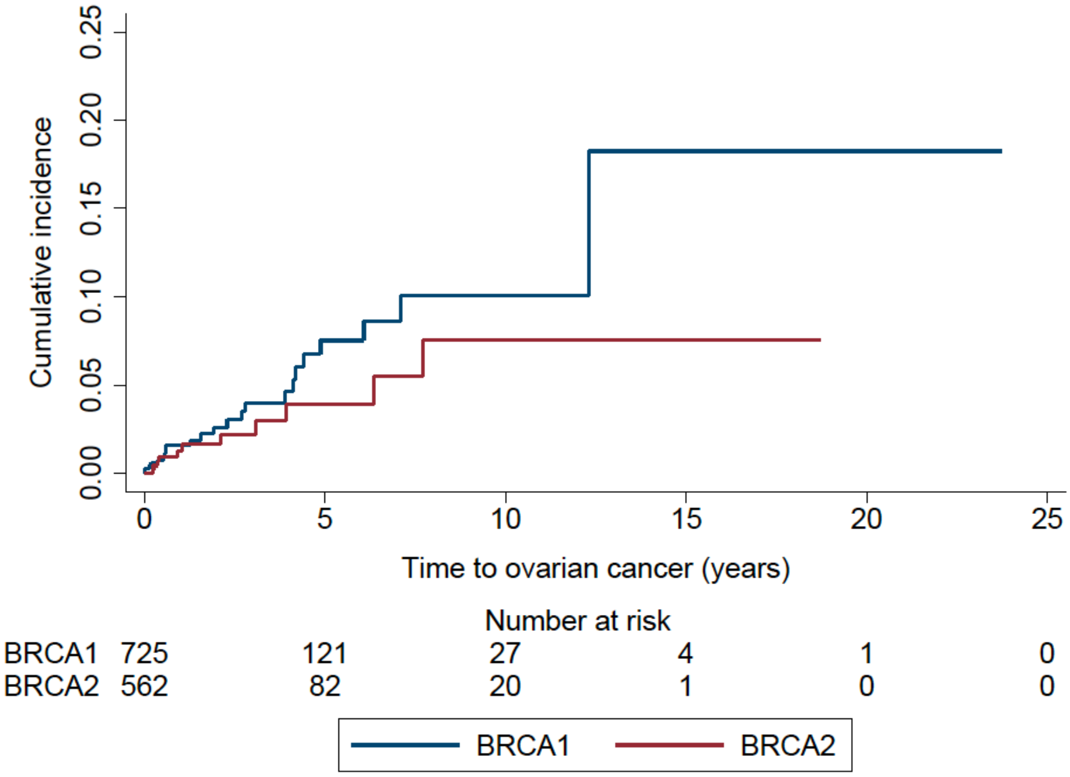

Supplementary Figure 1: Cumulative incidence of ovarian/peritoneal cancers in the *BRCA1* and *BRCA2* pathogenic variant carriers who did not undergo RRBSO during follow up.
